# A Case of Tracheobronchopathia Osteochondroplastica Diagnosed by Fiberoptic Bronchoscopy Upon Removal of an Aspirated Crown

**DOI:** 10.1155/DTE.7.63

**Published:** 2001

**Authors:** T. Matsuba, M. Komori, R. Matsunaga, K. Andoh, N. Hirota, N. Hara

**Affiliations:** 1 Research Institute for Diseases of the Chest Graduate School of Medical Sciences Kyushu University 3-1-1, Maedashi, Higashi-Ku Fukuoka 812-0082 Japan; 2 Nishi-Fukuoka Hospital 3-18-8, Ikinomatsubara Nishi-Ku Fukuoka 819-0555 Japan

## Abstract

Tracheobronchopathia osteochondroplastica (TO) is a relatively rare benign disease, around 300 and 130 cases have been reported in English and Japanese literature, respectively. Most of the cases have been diagnosed incidentally at autopsy. Due to the widespread use of fiberoptic bronchoscopy (FOB), the number of cases diagnosed upon examination is increasing. Here, we report a case of a 72 year-old man with a history of crown aspiration, who was diagnosed as TO upon removal of a foreign body using FOB. The diagnosis of TO and the removal of an aspirated crown by FOB are discussed.


*Diagnostic and Therapeutic Endoscopy*, Vol. 7, pp. 63–67
Reprints available directly from the publisher
Photocopying permitted by license only

© 2001 OPA (Overseas Publishers Association) N.V.
Published by license under
the Harwood Academic Publishers imprint,
part of Gordon and Breach Publishing,
a member of the Taylor & Francis Group.

### A Case of Tracheobronchopathia Osteochondroplastica Diagnosed by Fiberoptic Bronchoscopy upon Removal of an Aspirated Crown

T. MATSUBA[a,b,*], M. KOMORI[a,b], R. MATSUNAGA[b], K. ANDOH[b], N. HIROTA[b] and N. HARA[a]

[a]*Research Institute for Diseases of the Chest, Graduate School of Medical Sciences, Kyushu University, 3-1-1, Maedashi, Higashi-Ku, Fukuoka, 812-0082, Japan;* [b]*Nishi-Fukuoka Hospital, 3-18-8, Ikinomatsubara, Nishi-Ku, Fukuoka, 819-0555, Japan*

*(Received 24 August 2000; In final form 17 October 2000)*

**Tracheobronchopathia osteochondroplastica (TO) is a relatively rare benign disease, around 300 and 130 cases have been reported in English and Japanese literature, respectively. Most of the cases have been diagnosed incidentally at autopsy. Due to the widespread use of fiberoptic bronchoscopy (FOB), the number of cases diagnosed upon examination is increasing. Here, we report a case of a 72 year-old man with a history of crown aspiration, who was diagnosed as TO upon removal of a foreign body using FOB. The diagnosis of TO and the removal of an aspirated crown by FOB are discussed.**

*Keywords*: Aspiration, Dental crown, Foreign body aspiration, Tracheobronchopathia osteochondroplastica

*Abbreviations*: CT, chest-computerized tomography; FOB, fiberoptic bronchoscopy; TO, tracheobronchopathia osteochondroplastica

### INTRODUCTION

Tracheobronchopathia osteochondroplastica (TO) is a rare disorder which is characterized by accumulation of bony and cartilaginous nodules in the tracheal and bronchial mucosa. The disorder used to be an incidental finding at autopsy [1]. Due to the widespread use of fiberoptic bronchoscopy (FOB), the number of cases diagnosed upon examination is increasing [2]. Here, we report a case of a man with a history of crown aspiration who was diagnosed as having TO upon removal of the foreign body using FOB. TO, aspiration and removal of a foreign body in the airway are discussed as well.

* Corresponding author. Tel.: +81-92-642-5378. Fax: +81-92-642-5389. E-mail: tmatsuba@kokyu.med.kyushu-u.ac.jp.

### CASE REPORT

A 72-year-old man had a persistent dry cough, which began right after aspirating a crown during dental treatment. An initial chest X-ray (Fig. 1) showed an abnormal shadow (↑) in the right hilum which measured 16 × 11 mm and was presumed to be an aspirated crown. For confirmation and removal of the aspirated foreign body, he underwent an emergency bronchoscopic examination. Unexpectedly, endoscopic findings were suggestive of a malignant tumor of the trachea and he was admitted for further evaluation.

He had a history of bronchopneumonia 1-year prior to this procedure, but otherwise denied having any acute symptoms concerning chest disorders, recurrent respiratory infections, dyspnea on exertion, or hoarseness. A chest-computerized tomography (CT) scan demonstrated mild narrowing of the trachea (Fig. 2(A)) and both main bronchi (Fig. 2(B)). The affected bronchial wall was thickened and irregular with multiple calcified nodules and plaques of bony density. These lesions projected into the lumen and were most prominent in the anterior wall. Bronchoscopy, performed to confirm the aspirated foreign body, disclosed multiple hard, yellow-white papilla-like formations on the anterolateral wall of the trachea and main bronchi (Fig. 3↑), with an intact membranous portion. These lesions, 2–5 mm in diameter and densely distributed, either followed tracheal rings or were located between them. Mild narrowing of the trachea and bronchi were also noted. The mucosa overlaying the lesions appeared normal with no abnormality in the more distal bronchi.

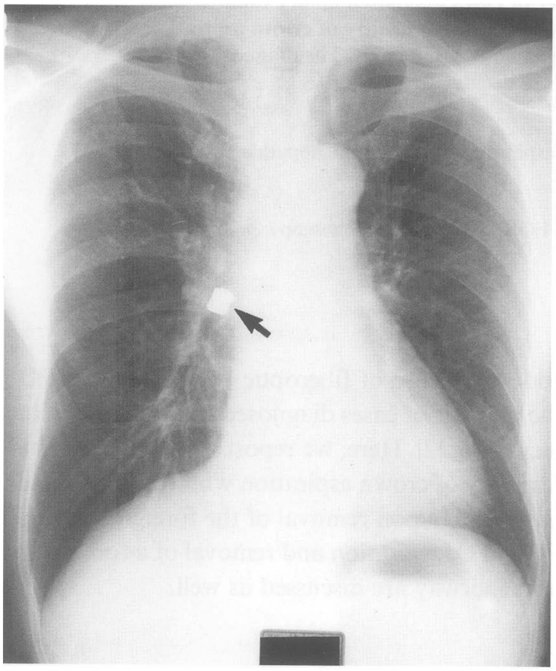

FIGURE 1 A chest X-ray film on presentation shows an abnormal shadow (arrow) suggestive of an aspirated crown at the right hilum.

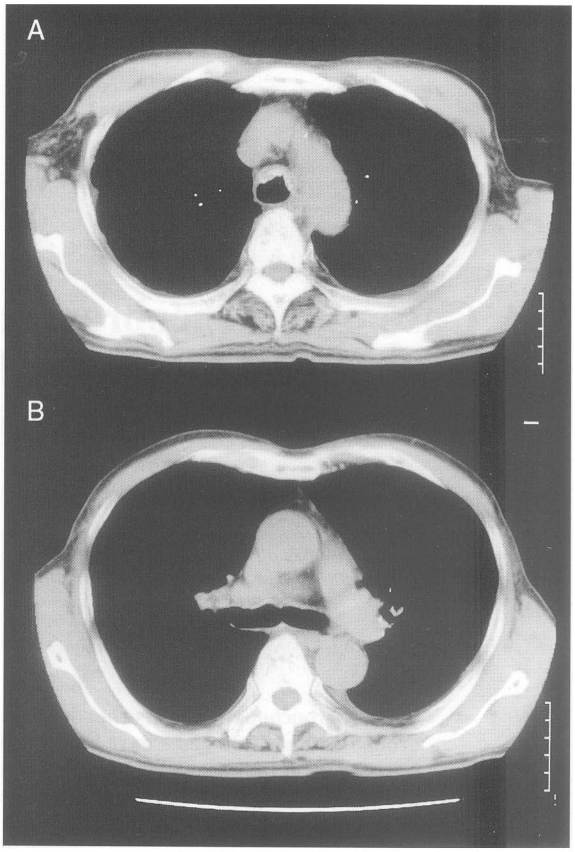

FIGURE 2 A chest CT scan on admission demonstrates the diffusely thickened and irregularly calcified surface of (A) the trachea and (B) both main bronchi. The anterolateral surface, but not the membranous portion of the trachea, was calcified.

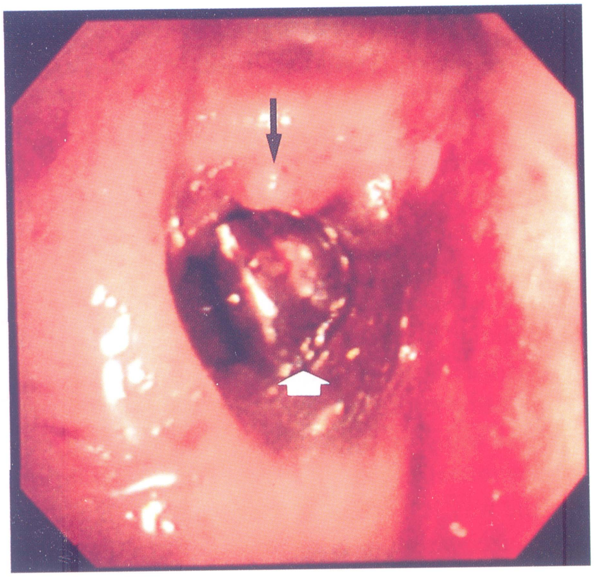

FIGURE 3 The aspirated crown (⇧) was lodged tightly at the trunchus intermedius. Yellow-white papilla-like formation (↑) was recognized in the bronchial wall.

The metallic foreign body (crown) was found to be lodged tightly at the trunchus intermedius (Fig. 3⇧). The bronchial mucosa around the foreign body showed redness and mild bleeding but was otherwise intact. Microscopy of biopsy specimens from the macroscopically altered mucosa showed irregular deposits of bone with fatty marrow tissue (Fig. 4 bottom) below the basement membrane of the squamous metaplastic epithelium (Fig. 4 top), consistent with the diagnosis of TO. During the bronchoscopic examination, both biopsy and foreign body forceps were used several times in an attempt to remove the aspirated crown. The slippery crown only permitted a temporary grasp for both forceps and we were unable to succeed with its removal. Therefore, closed basket forceps were introduced into the distal bronchus through a slight gap between the foreign body and the bronchial mucosa. The forceps were opened to cover and secure the foreign body which was carried above the vocal cords. After removal of the aspirated crown, the patient had an uneventful clinical course and was discharged after 5 days of admission.

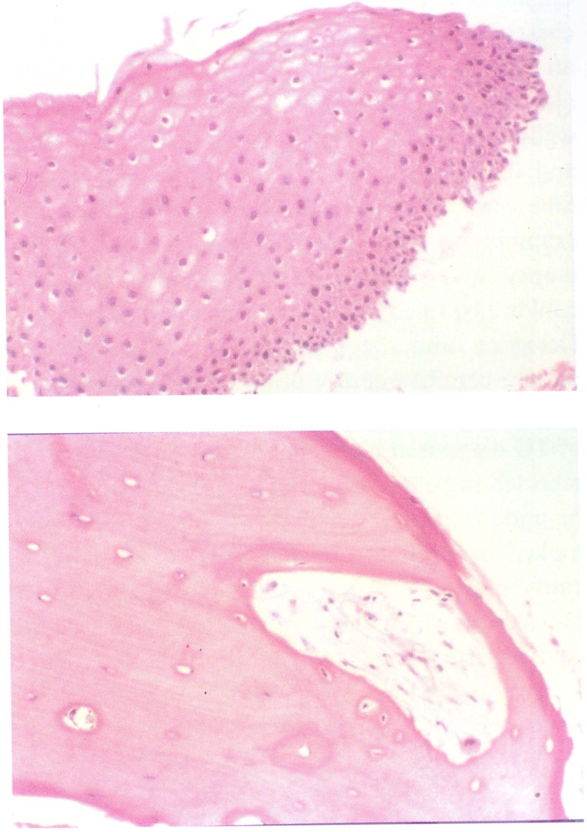

FIGURE 4 Microscopic findings of a nodule in the left main bronchus show the squamous metaplastic epithelium (top) and deposits of bone with fatty marrow tissue (bottom).

### DISCUSSION

TO is a rare disorder which was first described by Rokitansky in 1855 [3]. Since then, around 300 and 130 cases have been reported in English and Japanese literature, respectively. Most cases had been an incidental finding at autopsy, but an increasing number of cases have been diagnosed during a patient's lifetime by bronchoscopic examination [2], chest CT scanning [4,5], or during intubation [6] for other lung disorders. Cases associated with other lung disorders, such as mucoepidermoid carcinoma [7] and atypical mycobacteriosis [8], have been reported as well. To our knowledge, this is the first report of a case in which bronchoscopic examination was carried out after crown aspiration, eventually leading to the unexpected diagnosis of TO by bronchial biopsy. In our case, a chest CT scan, which had been performed during an episode of bronchopneumonia a year earlier, also showed thickened and irregular bronchial walls. However, these findings were overlooked and didn't give rise to a suspicion of TO. This time, the patient underwent a bronchoscopic examination before the CT scan, and a bronchial biopsy was performed, as a malignant tumor couldn't be ruled out from bronchoscopic findings. Detection and diagnosis of TO by a CT scan require careful interpretation of the findings with extensive knowledge of the disorder. The incidence of TO diagnosed by bronchoscopic examination is reported to be 0.02 [9] ~ 0.77% [10]. This appears to be underestimated as changes can easily be overlooked or misinterpreted [11]. Awareness of the entity may give a more accurate idea of its true prevalence, which can be higher. Many cases have been reported from Japan and Finland and there is a report suggesting a geographical difference in the occurrence of this disorder [10]. TO is usually found in subjects older than the age of 50 years regardless of gender [12]. Our case belongs to a slightly higher age group in view of previous reports. Symptoms are nonspecific which include cough, sputum, hemoptysis and fever. Twenty percent of the cases remain totally asymptomatic, as in ours. Although TO usually has a benign course, a high incidence (14%) for complicating a malignant tumor has been reported [2] and our case has been closely followed-up by our clinic.

Foreign-body aspiration into the airway is less common in adults than in children. The initial clues to foreign-body aspiration are usually obscure or indirect in adults, and only patients with a clear choking history will be suspected. According to recent reports from the United States [13] and China [14], the nature of aspirated foreign bodies differed between the two countries. However, the most commonly aspirated objects were food items, which accounted for 40.0% and 65.1% of all cases in the United States and China, respectively. Vegetables lead the list in the former and fish bones in the latter, reflecting different eating habits. Aspiration of a dental crown in adults accounted for 16.7% and 9.3% of aspirated foreign-body cases in the United States and China, respectively. Although there are no data available, the incidence of foreign-body aspiration in Japan is considered to be closer to that of China due to similar dietary habits.

When foreign-body aspiration into the lower airways is suspected, prompt examination with FOB should be performed [14]. In our hospital, FOB is used for conformation of foreign body as the first-line approach. The same is applied for its removal with the right choice of forceps. In this process, metallic and slippery nature of the crown and the way it is lodged are considered. The total FOB success rate in removal of a foreign body is reported as 82% [14]. The removal of the crown using regular foreign-body forceps is apt to be difficult [15]. In fact, forceps used for bronchoscopic removal of an aspirated crown include large biopsy forceps for laparoscopic cholecystectomy [15]. In another case, failure of the bronchoscopic retrieval procedure led to a tight lodgment of a foreign body in the airway too distal for access, which eventually required a thoracotomy [16]. Our bronchoscopic retrieval attempts with either biopsy or foreign-body forceps had failed, but finally succeeded using wire-basket forceps. In case the size of a foreign body is comparable to a dental crown and there is a slight gap between the airway, FOB would be useful in its removal with the choice of appropriate forceps such as wire basket forceps.

### *References*

[1] Prakash, U.B., McCullough, A.E., Edell, E.S. *et al.* Tracheopathia osteoplastica: familial occurrence. *Mayo Clin. Proc.* 1989; **64**: 1091–1096.

[2] Fujimoto, K., Kumabe, T., Fujitoh, H. *et al.* Two cases of Tracheobronchopathia osteochondroplastica and a review of 86 cases in the Japanese literature. *J. Jap. Soc. Bronchol.* 1991; **13**: 650–658.

[3] Rokitansky, K., Cited by Dalgaard, J.B.: Tracheopathia Chondroplastica. *Acta. Pathol. Microbiol. Scand.* 1947; **24**: 118–134.

[4] Park, S.S., Shin, D.H., Lee, D.H., *et al.* Tracheopathia osteoplastica simulating asthmatic symptoms. Diagnosis by bronchoscopy and computerized tomography. *Respiration* 1995; **62**: 43–45.

[5] Mariotta, S., Pallone, G., Pedicelli, G. *et al.* Spiral CT and endoscopic findings in a case of tracheobronchopathia osteochondroplastica. *J. Comput. Assist. Tomogr.* 1997; **21**: 418–420.

[6] Coetmeur, D., Bovyn, G., Leroux, P. *et al.* Tracheobronchopathia osteochondroplastica presenting at the time of a difficult intubation. *Respir. Med.* 1997; **91**: 496–498.

[7] Roggenbuck, C., Hau, T., de Wall, N. *et al.* Simultaneous occurrence of tracheobronchopathia osteochondroplastica and mucoepidermoid carcinoma. [German] *Chirurg* 1995; **66**: 231–234.

[8] Baugnee, P.E. and Delaunois, L.M. Mycobacterium avium-intracellulare associated with tracheobronchopathia osteochondroplastica. *Eur. Respir. J.* 1995; **8**: 180–182.

[9] van Nierop, M.A., Wagenaar, S.S., van den Bosch, J.M., *et al.* Tracheopathia osteochondroplastica. Report of four cases. *Eur. J. Respir. Dis.* 1983; **64**: 129–133.

[10] Vilkman, S. and Keistinen, T. Tracheobronchopathia osteochondroplastica. Report of a young man with severe disease and retrospective review of 18 cases. *Respiration* 1995; **62**: 151–154.

[11] Way, S.P.B. Tracheopathia osteoplastica. *J. Clin. Pathol.* 1967; **20**: 814–820.

[12] Akyol, M.U., Martin, A.A., Dhurandhar, N. *et al.* Tracheobronchopathia osteochondroplastica: a case report and a review of the literature. *Ear Nose Throat J.* 1993; **72**: 347–350.

[13] Limper, A.H. and Prakash, U.B.S. Tracheobronchial foreign bodies in adults. *Ann. Intern. Med.* 1990; **112**: 604–609.

[14] Chen, C.H., Lai, C.L., Tsai, T.T. *et al.* Foreign body aspiration into the lower airway in Chinese adults. *Chest* 1997; **112**: 129–133.

[15] Weiman, M.M., Weiman, D.S., Lingle, D.M. *et al.* Removal of an aspirated gold crown utilizing the laparoscopic biopsy forceps: a case report. *Quintessence Intern.* 1995; **26**: 211–213.

[16] Takanami, I., Shikata, J. and Morota, N. A case of bronchial foreign body which required thoracotomy. *J. Jap. Soc. Bronchol* 1991; **13**: 611–615.

